# Heterogeneous Multi-Robot System for Mapping Environmental Variables of Greenhouses

**DOI:** 10.3390/s16071018

**Published:** 2016-07-01

**Authors:** Juan Jesús Roldán, Pablo Garcia-Aunon, Mario Garzón, Jorge de León, Jaime del Cerro, Antonio Barrientos

**Affiliations:** Centre for Automation and Robotics (UPM-CSIC), José Gutiérrez Abascal, 2, Madrid 28006, Spain; pablogarciaaunon@etsii.upm.es (P.G.-A.); ma.garzon@upm.es (M.G.); jorge.deleon@upm.es (J.D.L.); j.cerro@upm.es (J.D.C.); antonio.barrientos@upm.es (A.B.)

**Keywords:** robotics, UGV, UAV, multi-robot, environmental monitoring, sensory system, griculture, greenhouse

## Abstract

The productivity of greenhouses highly depends on the environmental conditions of crops, such as temperature and humidity. The control and monitoring might need large sensor networks, and as a consequence, mobile sensory systems might be a more suitable solution. This paper describes the application of a heterogeneous robot team to monitor environmental variables of greenhouses. The multi-robot system includes both ground and aerial vehicles, looking to provide flexibility and improve performance. The multi-robot sensory system measures the temperature, humidity, luminosity and carbon dioxide concentration in the ground and at different heights. Nevertheless, these measurements can be complemented with other ones (e.g., the concentration of various gases or images of crops) without a considerable effort. Additionally, this work addresses some relevant challenges of multi-robot sensory systems, such as the mission planning and task allocation, the guidance, navigation and control of robots in greenhouses and the coordination among ground and aerial vehicles. This work has an eminently practical approach, and therefore, the system has been extensively tested both in simulations and field experiments.

## 1. Introduction

Agriculture and technology have evolved together, creating a close relationship throughout history. The technological advances of every period have been applied to this activity. Some examples are the use of animals in the neolithic period, the evolution of irrigation systems and the mechanization in the industrial revolution. Nowadays, the application of technologies, such as automation, robotics and computing, is transforming agriculture. Two examples are precision agriculture, which takes into account the spatial and temporal variability of crops to apply more targeted treatments, improving production and taking care of the environment [1], and technological greenhouse farming, which involves the control of the environment to provide the crops with optimal conditions for growth and maturation.

This paper is focused on greenhouse farming, which is especially receptive to new technologies. As pointed out before, the objective of greenhouses is to control the conditions of the crops, in order to increase the production and improve the quality. For this purpose, multiple developments of diverse sciences are applied in greenhouses, such as climate control (commonly, temperature and humidity sensors combined with heating and ventilation systems), soil preparation (from the simple addition of sand and clay to hydroponics) and other techniques (e.g., watering and nutrient supply, carbon dioxide enrichment and pollination with bees).

A review of the literature about greenhouses leads to the following automation proposals:

- Environmental monitoring and control [2,3,4,5]: These publications contain proposals about climate models and control systems. Most of them take the air temperature and humidity as target variables and the heating and ventilation systems as control variables. A detailed analysis of climate control and their requirements is addressed in the following section.
- Crop monitoring and supply [6,7,8]: These works analyze the requirements of crops and propose several systems for satisfying them. The products supplied include water, fertilizers and treatments to prevent infestations or curing diseases. Therefore, an efficient system is important not only for rationalizing these products, but also for taking care of the plants.
- Infestation and disease detection [9,10,11,12,13]: These papers show multiple proposals to detect both insect pests and plant diseases. In the first case, most of the works use computer vision techniques, sometimes with RGB images and other times with 3D images. In the second one, there are two kinds of methods: direct ones, when samples are taken in greenhouses and analyzed in laboratories, and indirect ones, when imaging techniques are used to detect the signs of diseases.
- Automatic planting and harvesting [14,15,16,17]: These publications propose automating the tasks that require more effort in the context of greenhouse farming: planting and harvesting. The proposals include different robots (rail and ground robots, for instance), sensors (e.g., various kinds of cameras and laser scanners) and effectors (e.g., manipulation and grasping systems).

Automating tasks in greenhouse farming has some advantages. First of all, automatic systems can be available all day and night, which is mandatory to correct environmental monitoring, climate control and failure detection. Additionally, these systems can reduce the exposure of human operators to hazardous environments, not only because of the working conditions (high temperature, humidity and solar radiation), but also because of some sensitive tasks (e.g., the application of some products to the crops). Finally, they can also provide both high quantity and quality of information, which allows new possibilities, such as local climate control and accurate product traceability.

However, the automation of these tasks also has some drawbacks to be resolved. One of these challenges is the work under harsh conditions, which may have impacts on the lifetime of robots, sensors, computers and actuators. Another one is the need for continued operation without stops or failures. This challenge can be addressed by designing flexible systems with redundant components, such as Wireless Sensor Networks (WSN) or multi-robot systems.

This paper proposes the use of a heterogeneous robot team for environmental monitoring in greenhouses. The team consists of two robots: an Unmanned Ground Vehicle (UGV) and an Unmanned Aerial Vehicle (UAV). Each robot provides the team with different capabilities: while the ground unit has robustness and autonomy, the aerial unit is agile, flexible and fast. Their features are taken into account when the mission planning and the task allocation are performed. Some collateral challenges, such as the Guidance, Control and Navigation (GNC) or the air-ground coordination, are considered, as well. All of the developments are validated both in simulations and with a series of field experiments performed in a greenhouse.

The paper is organized as follows: Chapter 2 summarizes the state of the art, focusing on greenhouse climate models and robotics applied to agriculture. Chapter 3 describes with detail all of the components of the multi-robot sensory system. Chapter 4 addresses the algorithms developed for mission planning, task allocation, navigation and air-ground coordination. Chapter 5 describes the field experiments performed for validating the components and algorithms of the multi-robot sensory system. Finally, Chapter 6 summarizes the conclusions of the paper and the proposals of future works.

## 2. State of the Art

Since the state of the art of automation and greenhouses is extensive, only the main contributions have been chosen and organized into two sections. Firstly, climate models and their variables are described, because they are relevant for the design of the sensory system. Secondly, previous works on robotics in greenhouses are reviewed, which is useful for the development of the robot fleet.

### 2.1. Greenhouse Environmental Variables

Greenhouses are complex systems for several reasons [18]: they are Multiple-Input and Multiple-Output (MIMO) systems, present nonlinear and coupled behaviors and are influenced by external disturbances and control system constraints.

In the literature, one can find several proposals of climate control in greenhouses [19]. Some examples of these strategies are conventional Proportional–Integral–Derivative (PID) control [18], adaptive control [20], fuzzy control [21], optimal control [22], predictive control [23] and robust control [24].

These control systems are based on different climate models of greenhouses. There are two approaches to obtain them: applying the equations of mass and energy flows, considering the greenhouse as the control volume [25], and identifying process functions through their inputs and outputs [26].

A review of climate models was performed in order to determine relevant variables. The results are shown in the following subsections, which correspond to the different kinds of variables.

#### 2.1.1. Input Variables

According to the literature, the following variables are used to control the system:

- Ventilation system [27]: These systems allow the air exchange between the greenhouse and its environment. This is important not only for controlling the greenhouse conditions (normally, they are used for cooling), but also for preventing the crop infestations and diseases. There are two main types of ventilation systems: natural ventilation and forced ventilation. In the first one, the warm air exits through the side and roof windows, and the control system manages their opening and closing. In the second one, the air exchange is controlled by the system using electric fans.
- Heating system [28]: These systems keep the temperature of the greenhouse in an appropriate range. If the temperature drops, for instance at night or in winter, the heating system compensates the potential heat losses. According to the layout, the system can be centralized or distributed; according to the operation, we can find systems that use heated fluids or electrical resistors.
- Fogging system [29]: Water is sprayed into the inside air, which increases the humidity and reduces the temperature. The control system can regulate both the activation times and the water flows.
- Shading screen [3]: Blinds are installed on the roof of some greenhouses and can regulate the incident solar radiation, preventing overheating inside the greenhouse in certain situations. These screens can be automatically deployed and collected by the control system. CO_2_ injection [3]: These systems supply carbon dioxide to the greenhouse, which has an influence on the photosynthesis of the plants, the gas flow being controllable.

#### 2.1.2. Output Variables

In addition, the following target variables are considered in the literature:

- Air temperature: The temperature control is key for crop growth and maturation. The consequences of excessive cooling or heating vary from the reduction of fruits size and quality to harvest losses. An optimal temperature control allows one to obtain off-season crops and even several harvests per year.
- Air humidity: The humidity control, together with the temperature control, is the base of greenhouse farming. An appropriate and balanced level of humidity is required to avoid plant diseases and insect pests.
- Solar radiation: The solar radiation heats the greenhouse during the day and the structure maintains the temperatures at night. This heat transfer is needed in cool seasons, whereas in warm seasons, it may damage the crops. CO_2_ concentration: As previously mentioned, the carbon dioxide concentration has an influence on the plant health and fruit maturation. More specifically, it is able to modify the internal characteristics of the plant (such us health and growth pace) and the external characteristics of the fruits (e.g., color and aroma) [30].

#### 2.1.3. External Disturbances

Finally, a list of disturbances considered by previous works is shown below:

- External temperature.
- External humidity.
- Wind speed.
- Wind direction.
- Solar radiation.
- External CO_2_ concentration.
- Cover temperature.
- Crop temperature.
- Soil temperature.

### 2.2. Robots in Agriculture

Most of the literature of environmental monitoring in greenhouses refers to WSNs [4,5,31,32,33,34]. In these works, sets of motes with temperature, humidity and luminosity sensors are deployed in the soil, crops and air. The objective of these systems is to measure the environmental conditions and to determine their spatial and temporal variation.

WSNs are flexible (they do not require a fixed architecture), modular (they can incorporate new devices) and fault tolerant (they still work when some motes fail) and have a low power consumption (i.e., the motes usually have a sleep mode). On the other hand, the network costs strongly depend on the covered area, since the motes are fixed. If the price of the sensors is high, this solution may be prohibitive.

Although WSNs have been widely implemented and present important advantages, the application of robots in greenhouse farming has grown in recent years. They can be used not only for environmental monitoring, but also for other tasks, such as crop spraying and fruit harvesting. Given that they can move the sensors and take measurements at any point of the greenhouse, mobile robots might improve the costs of WSNs.

Table 1 compares this paper with a diverse set of recent works, taking into consideration the system used, the application, the measured variables and the scenario. As shown, there are multiple proposals about using ground robots and one about using aerial robots in indoor agriculture. Additionally, several works use fleets with ground and aerial robots in outdoor agriculture. Nevertheless, the application of a robot team in the context of greenhouse farming can be considered as a novel contribution of this paper.

## 3. System Description

This section describes the multi-robot solution developed for the environmental monitoring of greenhouses. The section is organized as follows: Section 3.1 describes the type of greenhouses for which the system was designed. Section 3.2 addresses the robot fleet, including the features of robots and the performance of the team. Finally, Section 3.3 describes the selected sensors and their integration in the robots.

### 3.1. Greenhouse

Nowadays, there are over 700,000 *ha* of greenhouses worldwide, and this figure grows every year. The main world regions in greenhouse farming are China and the Mediterranean Basin, while the largest agglomeration of greenhouses with more than 30,000 *ha* is in Almería (Andalusia, Spain) [44].

This work is focused on the typical greenhouses of this last region, which have an average surface area of 6200 m^2^ [45]. The main crops in terms of production and surface area are tomato, pepper, cucumber, courgette, watermelon, melon, aubergine and bean. These crops grow in prepared soils (adding clay, organic matter and sand) or directly in controlled substrates (e.g., hydroponic crops). The structures of greenhouses in Almería are usually simpler than in other regions. They consist of several pillars manufactured with iron tubes or profiles, which support the wire mesh with a square pattern, and the plastics that cover all roofs and sides. More details of the common exploitations in Almería can be found in periodic surveys [46].

Greenhouses are closed facilities with high occupancy, thanks to the efficient use of the available space. They have a structured layout (i.e., they consist of a series of crop lines and corridors), but they also have irregular elements (e.g., the plants are planted in regular places, but grow irregularly). This factor should be considered not only when selecting the robots, but also when defining the strategies for path planning, localization and path following.

**Figure 1:**
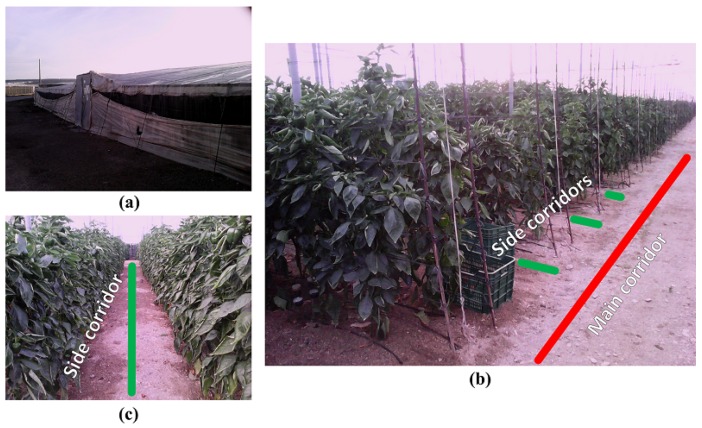
A typical greenhouse of Almería from multiple perspectives: (**a**) Outside; (**b**) Main corridor; and (**c**) Side corridor.

As can be seen in Figure 1, these greenhouses often present a front side with one or more doors that can be used for machinery, some main corridors with a width of around 2 m and some side corridors with a width of around 1 m.

### 3.2. Robot Team

As stated above, a multi-robot sensory system was designed, developed and tested. Some potential contributions of using a robot team instead of a single robot are the following:

- Effectiveness: A robot team is obviously more effective than a single robot, because it has more resources to perform the same tasks. For instance, if a robot covers an area in a certain time, multiple robots will necessarily cover multiple subareas, spending less time.
- Efficiency: The robot fleet is also more efficient than the single robots, since the allocation of the tasks to the robots can be optimized. For example, an aerial unit will cover more surface than a ground unit in some tasks, such as surveillance or monitoring.
- Flexibility: A robot fleet is more flexible than a single robot, because it is able to adapt to different scenarios by only changing the assignation of tasks.
- Fault tolerance: Using a robot team instead of a single robot reduces the impact of failures and contingencies; if one of the robots fails, the rest can take over its duties.

**Figure 2:**
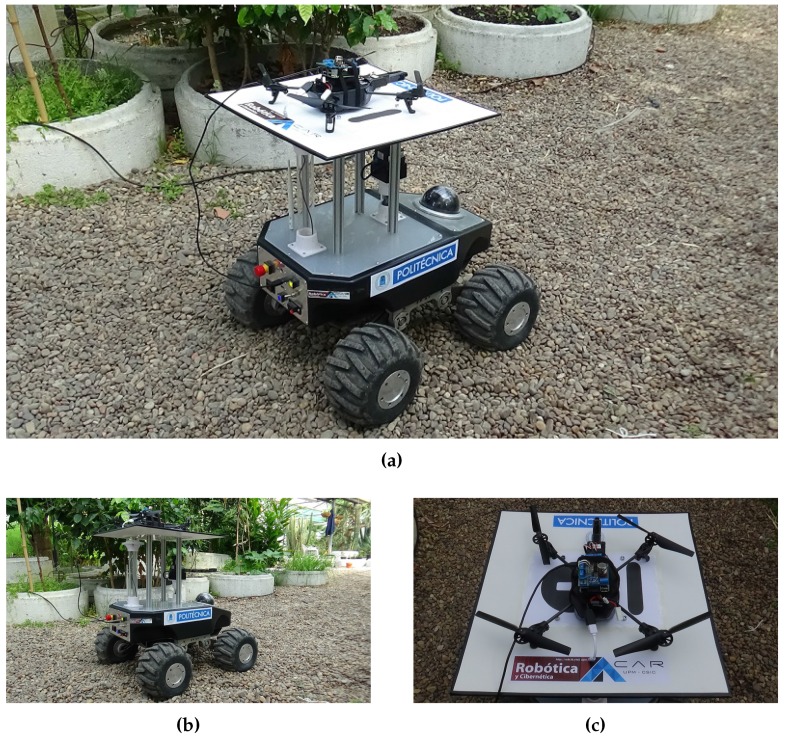
(**a**) Air-ground robot team; (**b**) Ground robot: Robotnik Summit XL; (**c**) Aerial robot: Parrot AR.Drone.

In previous works, we used an aerial robot [40] and a ground robot [43] to measure the environmental variables of greenhouses. In the first case, a mini-UAV was used to measure air temperature and humidity, luminosity and carbon dioxide concentration, whereas in the second work, a medium UGV was used to measure ground temperature and moisture. Additionally, we researched the air-ground cooperation applied to obstacle detection and mapping [47]. The proposal of this paper is to apply a team made up of these two robots. The robots are shown in Figure 2, and their main features are described below.

#### 3.2.1. Ground Robot

The UGV used as ground sensor platform is Robotnik Summit XL [48]. It is a wheeled robot with a medium size (722 mm × 610 mm × 392 mm) and weight (45 kg) and a remarkable load capacity (20 kg). It has four wheels with four motors, so it is able to rotate in place. It is controlled by an embedded computer with Linux and the Robot Operating System (ROS).

This robot can use the encoders of its wheels, the Inertial Measurement Unit (IMU) and the Global Navigation Satellite System (GNSS), for navigating in the greenhouse. It can also use two linear laser scanners (one of 270° on the robot to measure at around 65 cm and another of 120° down it to measure at around 20 cm) to detect and avoid the possible obstacles in the corridors (e.g., agricultural tools and plant branches). Additionally, it is equipped with a pan-tilt-zoom camera that can be useful in certain inspection tasks.

Finally, this UGV can reach a maximum speed of 3 m/s and has an autonomy between 5 h (continuous motion) and 20 h (standard laboratory use).

#### 3.2.2. Aerial Robot

The UAV used as an aerial sensor platform is the Parrot AR.Drone 2.0 [49]. It is a commercial quadrotor with a size of 525 mm × 515 mm × 120 mm and a weight between 380 and 420 g, depending on whether it is equipped with the hull for outdoor or indoor flights.

This small quadcopter is electrically powered and has autonomy of around 18 min with 1500-mAh batteries. It utilizes its four propellers to stabilize, change roll (going to the left or right), pitch (going forward or backward) or yaw (rotating in place) and to move vertically (ascending or descending).

This quadrotor is controlled by an ARM processor and can connect to other devices via WiFi networks. It has an IMU, a ultrasonic altimeter and two cameras (one in front and another under the UAV). It can reach speeds over 5 m/s.

#### 3.2.3. Team Strategy

As previously pointed out, each robot provides the team with different capabilities according to its features, which are summarized in Table 2. In this manner, the UGV contributes with high autonomy (it can continuously patrol the greenhouse for up to 5 h), robustness (it has high resistance and load capacity) and fault tolerance (the consequences of a failure are less harmful). On the other hand, the UAV provides the team with three-dimensional movement (it is able to place the sensors at any point of 3D space), agility (it can easily overcome situations where obstacles on the ground are blocking the path) and speed (it is able to cover the same path consuming less time).

The proposal of this paper is to take advantage of the differences between the UGV and UAV and improve the performance of the whole team. The multi-robot system works as follows: the UGV carries the UAV on a platform while it develops its tasks, and when it is required, the UAV takes-off, performs some tasks and lands on the UGV. We assume that the UAV can charge its batteries while it is coupled to the UGV, and the UGV can charge its owns when it is stopped at certain locations of the greenhouse. Therefore, the team strategy seeks to combine the robustness and autonomy of the UGV in continuous work with the agility and speed of the UAV in occasional interventions.

The UAV should intervene in three cases: first, when the UGV has low battery and cannot perform a complete tour around the greenhouse; second, when the UGV detects an obstacle in the corridor and cannot continue the planned path; and third, when the sensors measure unusual values and it can fly around this location to investigate the cause. For this purpose, a platform has been designed, developed and installed on the UGV for the transport, take-off and landing of the UAV.

### 3.3. Sensors

A set of greenhouse models contained in the literature were studied in previous chapters. The climate control takes into account four controlled variables (air temperature and humidity, solar radiation and CO_2_ concentration) and a series of disturbance variables (e.g., external temperature and humidity, wind speed and direction and soil temperature and humidity).

In this work, the multi-robot mission aims to measure temperature, humidity, luminosity and carbon dioxide concentration in the greenhouse. Nevertheless, other variable,s such as the concentration of other gases, could be obtained by adding the adequate sensors, keeping always in mind the limited load capacity and power consumption of the aerial vehicle. Additionally, autonomous visual inspection can be performed by using the cameras of both robots.

The RHT03 sensor was selected to simultaneously determine air temperature and humidity, while the TSL2561 sensor was chosen to measure luminosity and the MG811 sensor to measure CO_2_ concentration. The features of these sensors are collected in Table 3. These sensors are installed on the UAV, and therefore, the measurements refer to both robots when they are together and to the UAV when they are separated.

**Figure 3:**
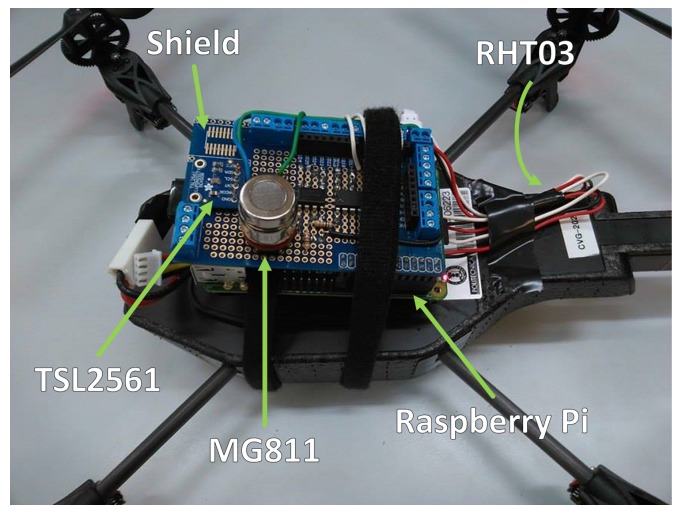
Integration of RHT03 temperature and humidity, TSL2561 luminosity and MG811 carbon dioxide sensors in Parrot AR.Drone 2.0 through Raspberry Pi and shield.

As shown in Figure 3, the integration of these sensors in the UAV was performed by using a Raspberry Pi. The RHT03 sensor is directly connected to a digital pin; the TSL2561 sensor is connected via I2C (Inter-Integrated-Circuit) using SDA (Serial Data Line) and SCL (Serial Clock Line) pins; and the MG811 sensor is connected to a digital pin through an ADC (Analog to Digital Converter). A program developed under ROS Hydro and Raspbian Wheezy reads the measurements of the sensors and sends them to the whole system.

## 4. Algorithms

**Figure 4:**
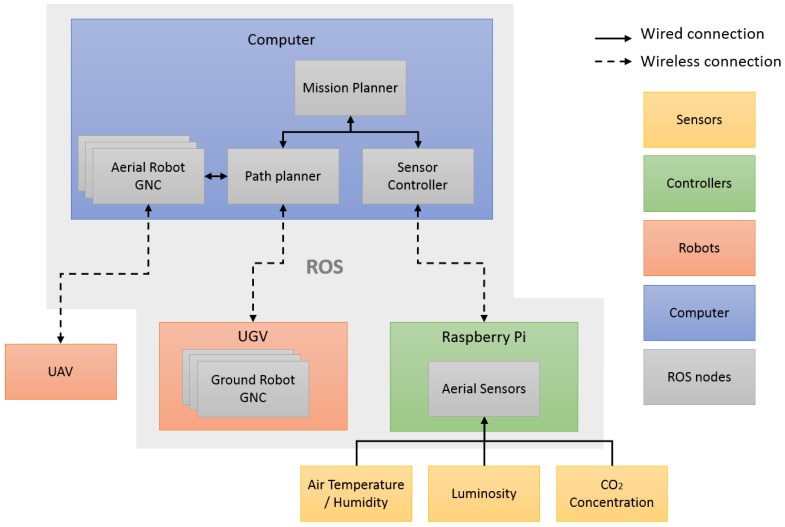
ROS architecture with robots, sensors, controllers and the central computer.

This chapter addresses the algorithms developed for controlling and monitoring the robot team. All of these algorithms work under the ROS framework, which allows the integration of sensory and navigation algorithms and the coordination between aerial and ground robots. The architecture presented in Figure 4 summarizes the integration of the robots and sensors. The algorithms for mission planning are explained in Section 4.1, whereas the algorithms for robot navigation are described in Section 4.2.

### 4.1. Mission Control and Monitoring

The robot team shall cover the greenhouse completely and periodically to monitor the environmental variables. The proposal of this paper is to perform a model-based mission control and monitoring. This model can be obtained through the experience of previous missions, and the process to obtain it is described below.

**Figure 5:**
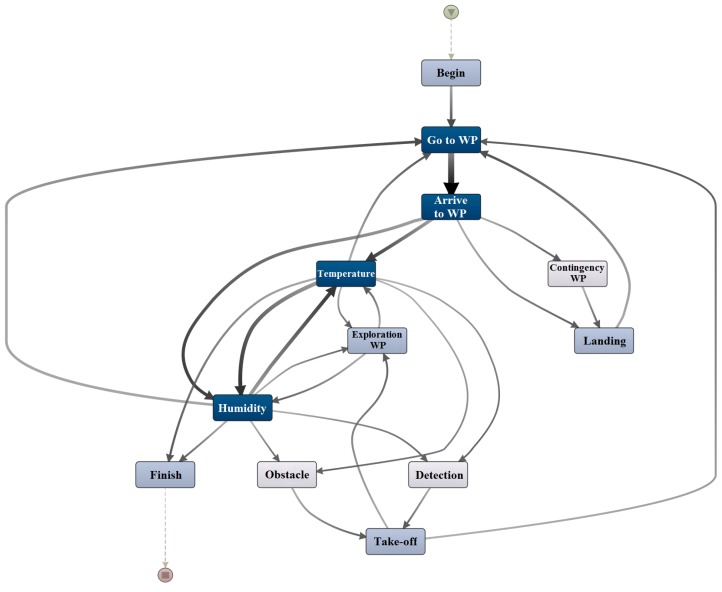
Mission event log.

A simulator was used to reproduce the multi-robot mission with diverse scenarios (i.e., greenhouses of different sizes and layouts) and contingencies (e.g., presence of obstacles in the robot paths). Some theoretical specifications and experimental measurements of the robots were introduced in the simulator. This simulator is used for testing the missions before their application in the real greenhouse and is described with more details in Section 5.1. Additionally, this simulator generates event logs of the missions, which include the events, their date and time and the agents that perform them. A fragment of the event log is shown in Table 4, while the whole event log is depicted in Figure 5.

**Figure 6:**
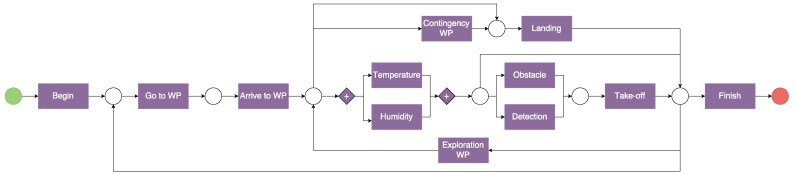
Mission model.

Process mining [50] is an emerging discipline that addresses the analysis of processes through event logs. It involves the discovery of models through event logs, the reproduction of models to obtain event logs and the subsequent conformance checking between models and event logs. The discovery algorithms [51] generate models, such as Petri nets, from the event logs. It should be noted that the event logs only contain relations of precedence between events, while the models are able to manage relations of causality or parallelism between them. In this paper, we applied the Inductive Miner algorithm [52] that is implemented in the *PROM 6.5.1* toolkit to obtain the model of Figure 6.

The model integrates the actions of robots, the environment and operators, which allows the management of complex missions involving multiple agents. This model is used to control the robot paths and payload actions (e.g., sensor measurements), and its level of detail can be adjusted according to the necessity. Finally, the model can be improved during the operation, just adding the new cases to the event log and applying again the discovery algorithm.

### 4.2. Guidance, Navigation and Control

The GNC of robots in greenhouses is a challenge to be faced with this multi-robot system. This objective encompasses different tasks (i.e., path planning, localization, path following, etc.) for both ground and aerial robots.

First of all, the mapping of environmental variables requires the coverage of the greenhouse. The literature contains multiple strategies for coverage path planning [53]. Back and forth motions and spiral paths are suitable alternatives in unknown open fields [54]. Given that greenhouses are highly structured facilities, a suitable coverage method must take their distribution into account. The optimal path should pass through the maximum number of plants, covering the minimum distance and spending the minimum time. In this work, the back and forth strategy is selected considering the objectives of environmental monitoring and the layout of greenhouses.

**Figure 7:**
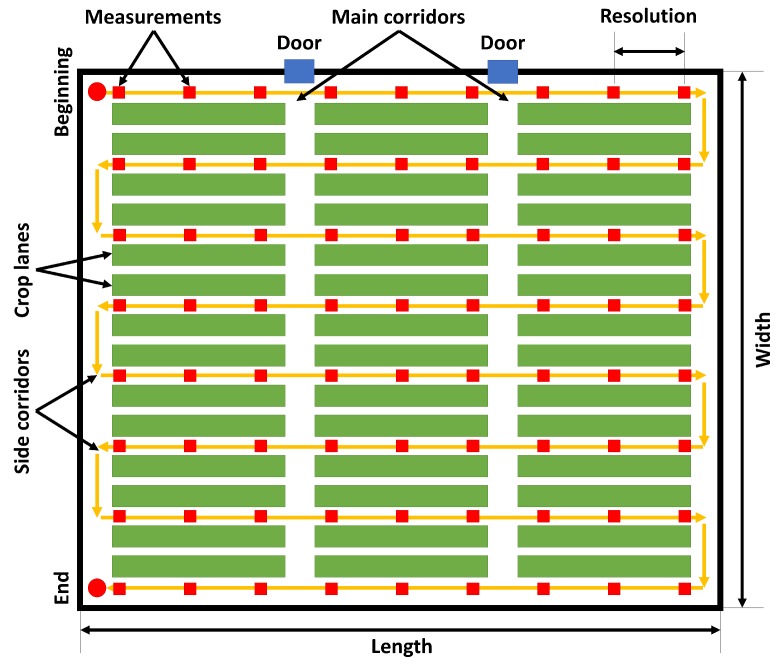
Coverage path planning for the robot team in the greenhouse.

Figure 7 shows an example of this coverage path in a greenhouse. As can be seen, the path goes through all of the side corridors and passes along all of the crop lanes. The beginning and the end are located in two corners of the greenhouse, where the required infrastructure to store and charge the robots can be installed. Furthermore, the route features depend on the size of the greenhouse (number of corridors) and the resolution of monitoring (measure points).

On the other hand, the robots must be able to localize themselves and travel inside the greenhouse. The literature mainly covers two basic paradigms for robot navigation in agricultural applications [55]: computer vision, taking advantage of crop features (e.g., crop lines and plant contours) or adding visual markers (e.g., ground lines and image codes) and global positioning, using GNSS to locate the robots in the map [9].

More specifically, in the context of greenhouse farming, various techniques have been proposed, implemented and tested. For instance, González et al. [56] combined deliberative map-based algorithms to create safe paths through the greenhouse with reactive sensor-feedback algorithms to move the robots through the corridors. Other proposals include the use of distance sensors to keep the robots in the center of the corridors [57] or the use of cameras to track lines printed on the ground [8]. The following subsections explain the selected navigation systems for the ground and aerial robots.

#### 4.2.1. Ground Robot

**Figure 8:**
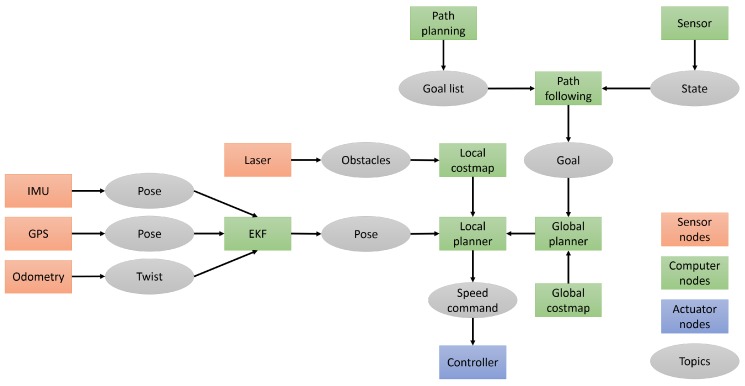
Architecture of the multi-robot sensory system.

The GNC algorithm for the ground robot is based on the sensors (Odometry, IMU, GNSS and laser scanner) and the ROS navigation stack [58]. The ROS architecture is shown in Figure 8 and described in the following paragraphs.

Three measurements of the robot pose (i.e., position and orientation) are obtained through odometry, IMU and GNSS. These measurements present uncertainty because of the inherent noises and biases of the sensors. An extended Kalman filter (EKF) is used to integrate the measured poses and to estimate the global pose. This filter uses a motion model for linking the data of sensors with the position of robot and a Bayesian-based fusion technique to combine the measurements in a probability density function. Additionally, the laser scanners provide the robot with information about the obstacles around it. In the greenhouse, this sensor is able to detect the corridors and to determine the robot position, which is useful to compensate the localization errors and to perform a safe navigation.

The ROS navigation stack is configured to perform an Augmented Monte Carlo Localization (AMCL). This algorithm estimates the position and location of the robot taking into account the map and the sensor data by using a particle filter [59]. It combines a global path planner, which finds the optimal path between the current position and the goal, and a local planner, which generates the short-term trajectory taking into account the robot kinematics and the obstacles. The global planner manages a global costmap with the knowledge of the greenhouse previously acquired to the mission, while the local planner builds local costmaps that include the static and dynamic obstacles. These costmaps are used to distinguish free and occupied areas at the time of path planning. The ROS navigation stack generates speed commands to the robot controller, which manages the voltage of the motors and the speed of the wheels.

The path planning node generates the back and forth path to cover the greenhouse. This path can be modified during the mission when some conditions are satisfied. For instance, if an obstacle blocks the corridor or if the measurements are outside of ordinary values, the UGV must change the plan, and the UAV should take part in the mission. Besides, the path following node sends the goals to the navigation stack one by one. This node controls the stop and start in the measure points.

#### 4.2.2. Aerial Robot

As previously explained, the aerial robot intervenes in three main situations:

- When the UGV has low autonomy, the UAV should continue the mission.
- When the UGV finds an obstacle and cannot avoid it, the UAV must continue the route.
- When the UGV gets anomalous measurements, the UAV must move around looking for the source.

Therefore, the UAV must move through the corridors in the first two cases and travel along grids around the UGV in the third case.

Small quadcopters are popular robots for research and education purposes [49,60], since they are low-cost platforms that allow developing diverse algorithms of GNC, sensor fusion, computer vision, etc. Specifically, the literature regarding the navigation of mini-UAVs is extensive and can be split into outdoor and indoor scenarios.

Most of the proposals regarding outdoor flights are based on GNSS [60,61]. They take into account the readings of other sensors, for instance IMUs, and use methods for fusing them, such as a Kalman filter. In some cases, computer vision techniques have been also applied to improve the pose estimation.

However, flying in greenhouses presents some characteristics that complicate the application of algorithms developed for outdoor flights. On the one hand, the GPS signal may have lower quality, due to the influence of the plastic covers and metallic structures. On the other hand, the localization requires more accuracy, in order to navigate along narrow corridors between the plants.

The proposals about indoor flights [49,62,63,64,65,66,67,68] are more interesting from the perspective of a possible application in greenhouses.

Engel et al. [65] proposed a combination of a Simultaneous Location and Mapping (SLAM) for visual odometry using a front camera, an EKF for fusing the readings of the IMU and the altimeter and a PID control for stabilization and navigation. They tested the algorithms in diverse environments, such as offices, kitchens and parking lots. They obtained a position error while hovering between 4.9 and 18.0 cm according to the scenario. Unfortunately, greenhouses are not as regular as these scenarios, which may increase the errors in localization.

Tomic et al. [64] used a quadcopter equipped with an IMU, a laser and four cameras: two stereo, one front and one up camera. They estimated the odometry of the robot through the stereo camera and the laser and combine this odometry with the attitude of the IMU by means of an EKF. Furthermore, they use the other cameras to recognize the environment and perform mission control and monitoring. To carry all of these sensors, a heavier and more powerful quadrotor must be used. Although the navigation performance may improve, the stronger airflow could damage the crops.

Lamberti et al. [67] proposed a two-vision-based pose estimation method, which consists of relative and global systems. The relative system uses the vertical camera for tracking the movement from frame to frame and to estimate the quadrotor pose. The global system uses the horizontal camera to detect periodic markers and compensate the deviations in the estimations. This navigation system is adapted to regular scenarios, and therefore, some changes are required in the greenhouse.

In this paper, we teleoperate the quadcopter with a joypad and the support of a control algorithm based on visual odometry. Nevertheless, in future works, the different alternatives for autonomous flight will be tested in the greenhouse, in order to determine their performance and apply the most appropriate one.

## 5. Experiments, Results and Discussion

This section collects the experiments performed to validate the cooperative system and determine its performance. The section is organized as follows: Section 5.1 addresses the simulations that estimate the system performance in different types of greenhouses. Section 5.2 presents the experiments performed in a real greenhouse to build maps of environmental variables.

### 5.1. Simulations

A complete set of simulations was carried out in order to estimate the performance of the system. On the one hand, these simulations allow performing a mission analysis to obtain the models shown in Section 4.1. On the other hand, they provide valuable information about the system, such as the time and battery required to perform the complete route.

**Figure 9:**
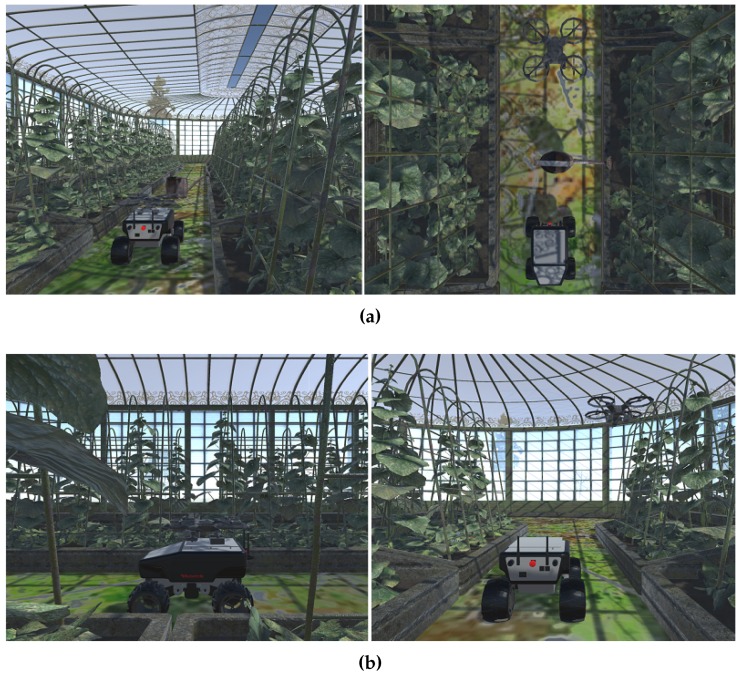
Two situations considered in the simulations: (**a**) The UGV cannot avoid an obstacle, so the UAV continues the route, and they meet again in the next corridor; (**b**) The UGV detects an anomaly in the environmental variables, and the UAV moves around looking for the source.

For this purpose, a mission simulator was developed under the *Unity3D 5.2.1*game engine. As shown in Figure 9, this simulator reproduces the missions with similar robots (UGV and UAV) in a realistic scenario (a greenhouse in our case). Since the aim of the simulator is to obtain general information about mission performance and not particular information about the robot paths, the focus is on the multi-robot coordination to accomplish the mission, rather than their dynamics and kinematics.

The first simulations looked to determine the frequency at which the robot team can perform a complete coverage of the greenhouse. In this respect, they were developed in greenhouses of different sizes (from 3600–10,000 m^2^) around the average surface area in Almería (6200 m^2^). Two strategies were compared: firstly, the UGV covering the greenhouse alone and, secondly, the UGV covering most of the greenhouse and the UAV covering some corridors, depending on its autonomy. The results are collected in Table 5 and analyzed below.

The time spent by a single robot in visiting some measurement points can be split into the time to move along the paths, to turn and to measure (Equation (1)). The first one (Equation (2)) can be estimated through the number of points ($n_{p}$), the distance between them ($d_{p}$) and the robot average speed (*v*), whereas the second one (Equation (3)) can be computed through the number of corridors ($n_{c}$), the distance between them ($d_{c}$) and the robot average speed (*v*). Finally, the third one (Equation (4)) depends on the response times of the different sensors ($T_{r}$).

(1) $$T_{robot}=T_{move}+T_{turn}+T_{meas}$$

(2) $$T_{move}=\frac{\left(n_{p}-1\right)*d_{p}}{v}$$

(3) $$T_{turn}=\frac{\left(n_{c}-1\right)*d_{c}}{v}$$

(4) $$T_{meas}=max\left(T_{r}\right)$$

On the other hand, the time spent by the complete robot team in the coverage of the greenhouse can be divided into the ground robot time, the aerial robot time and the autonomous take-off and landing time (Equation (5)). Additionally, the global time should include not only the coverage time, but also the preparation time for the next cycle (Equation (6)). This preparation time can be computed as the time required to charge the batteries of the robots.

(5) $$T_{team}=T_{UGV}+T_{UAV}+T_{atol}$$

(6) $$T=T_{team}+T_{prep}$$

**Figure 10:**
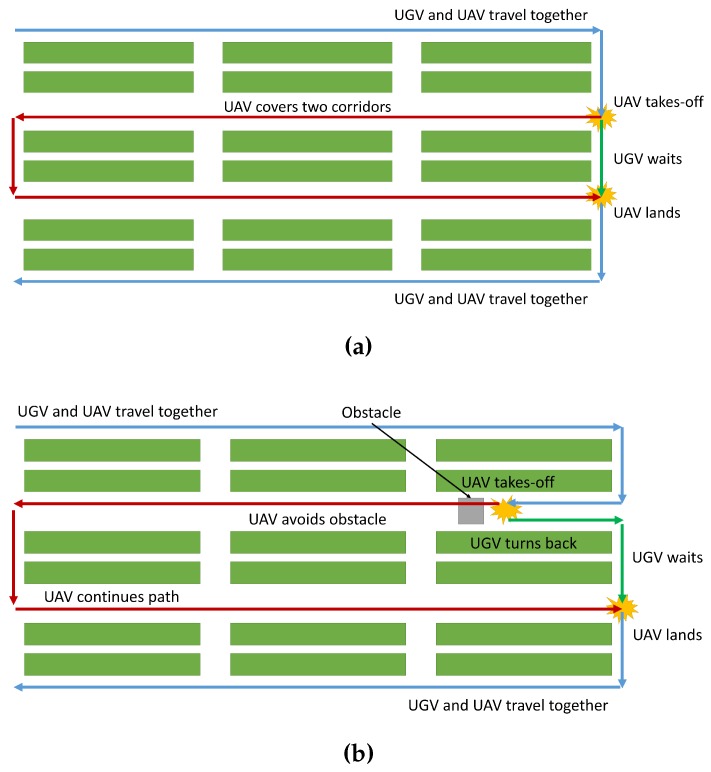
Application of the team strategy in simulations: (**a**) First simulation: the UAV covers two corridors, and the UGV covers the rest; (**b**) Second simulation: the UAV avoids the obstacle, while the UGV turns back.

Let’s see an example: in a greenhouse of 6400 m^2^ (80 m × 80 m) with a resolution of 10 m, the UGV can cover six corridors and the UAV two, as shown in Figure 10a. The UGV spends 2104 s and 11.69% of the battery, while the UAV spends 454 s and 43.80% of the battery. The complete coverage time is 2558 s, and the preparation time is 841 s, resulting in a global time of 3400 s (57 min). As shown in Table 5, the use of the team implies an improvement of 11.05% over using a single ground robot in terms of global time. This fact is due to the UGV spending less battery and the UAV being faster than the UGV.

The second simulation was conducted to compute the performance of the team when there are obstacles in the greenhouse. For instance, if an object is blocking a corridor of the greenhouse, the UGV must come back through the other side of the corridor. However, the team can overcome this situation by avoiding the obstacle with the UAV and meeting both robots in the following corridor, as shown in Figure 10b.

The simulations were again developed in greenhouses of different shapes and sizes, and the results are collected in Table 6. The UAV contributes to the UGV in these scenarios with an improvement of 19.67% in terms of global time.

In summary, the robot team improves the performance of the ground robot between 8% and 23% depending on the scenario. This improvement is more significant in small-sized greenhouses and in the presence of obstacles. Note that, in any case, the global time varies between 30 min and 1.5 h depending on the size of the greenhouse. This implies that the use of a team with more units may be required in large greenhouses. It should be remarked that these results have been obtained with certain robots and may change if robots with different speeds or autonomies are employed.

### 5.2. Experiments

**Figure 11:**
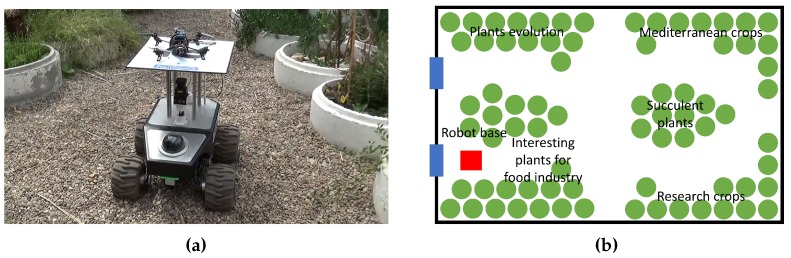
Experiments: (**a**) robot team; (**b**) greenhouse layout.

These experiments were performed in an educational greenhouse of the Agricultural School of the Technical University of Madrid to validate the work of the multi-robot sensory system. This greenhouse was designed to preserve and show plants from various regions of the world. Therefore, the layout of this greenhouse is less regular than the distribution of productive greenhouses, as can be seen in Figure 11. In addition, the presence of different crops that require different conditions causes a spatial variation of the environmental variables. In this work, both effects are going to be verified by the system: the navigation in an irregular facility and the high spatial variability.

**Figure 12:**
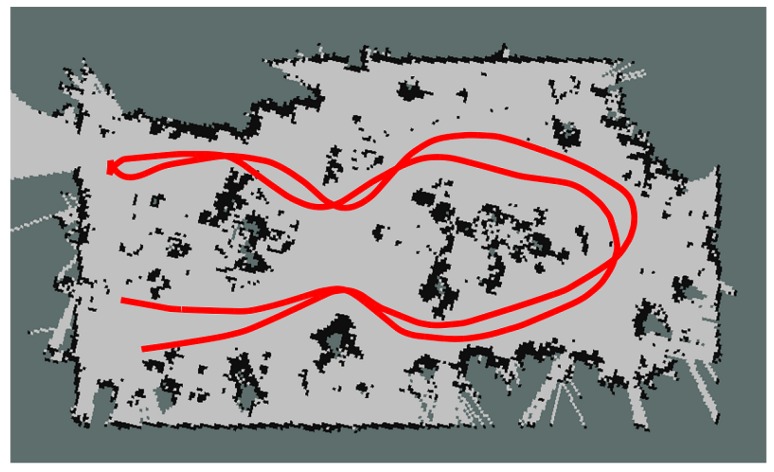
Map of the greenhouse used in the experiments. In light gray, the free spaces; in black, obstacles; in dark gray, unknown spaces; and in red, the robot path.

Firstly, the ground robot was teleoperated following a path inside the greenhouse, in order to build a map with the SLAM algorithm. This algorithm uses two references to obtain the location of the robot: one is the fusion of odometry and IMU measurements with the EKF and the other the readings of the lasers. In this manner, the algorithm is able to determine the robot pose while it is building the map. The resulting map is shown in Figure 12, where the light areas are the corridors and the dark areas are plants.

**Figure 13:**
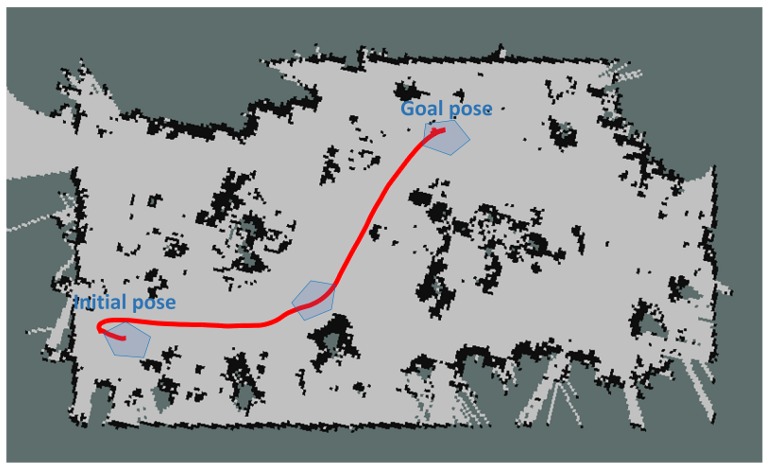
An example of autonomous movement between the starting point and the goal position.

Additionally, the autonomous navigation of the ground robot in the greenhouse was tested. For this purpose, we placed the robot in different poses, and different goals were sent. Figure 13 shows one of these movements: the robot had to rotate in a reduced place, advance to the destination avoiding the plants and rotate again to reach the desired orientation. The navigation was performed using the AMCL algorithm with the previously-obtained map. Again, odometry and the IMU were used to estimate the relative location, whereas the two lasers and the map to correct the deviations.

**Figure 14:**
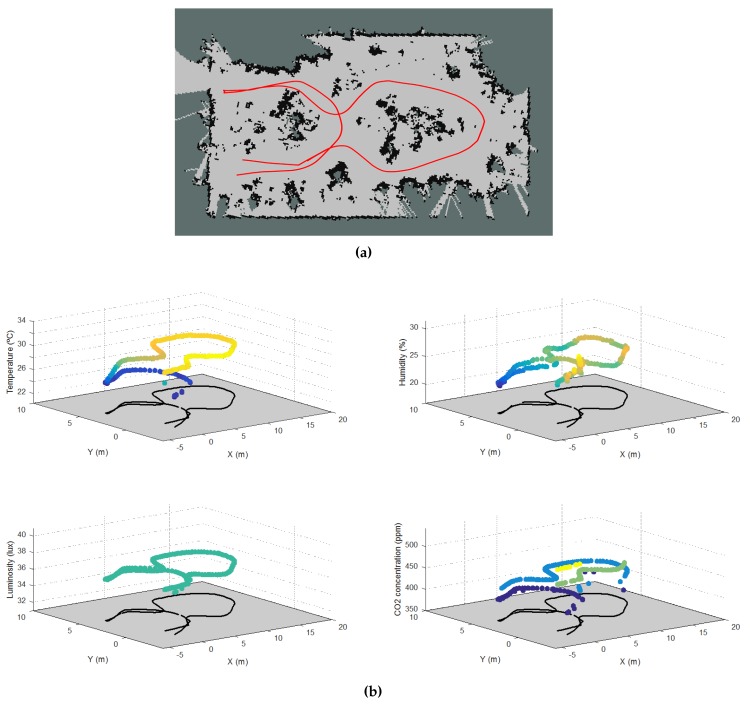
Mapping of the environmental variables of greenhouses: (**a**) Ground robot path in the greenhouse; (**b**) Distribution of temperature, humidity, luminosity and CO${}_{2}$ concentration in the route.

Afterwards, the ground robot was used to cover the greenhouse and to build maps of environmental variables. Figure 14a depicts the path taken by the robot in the greenhouse, which passes at least once through each corridor. Figure 14b shows the four maps of the four environmental variables: temperature, humidity, luminosity and carbon dioxide concentration. As can be seen, there is a difference between the measurements at the beginning and the end of 1.7 °C that probably can be explained by the changes along time. Other variables, such as the humidity and the CO_2_ concentration, show a spatial variability, reaching higher values in some areas and lower values in other ones without following a time pattern. Finally, the luminosity is practically constant during the path of the robot in the greenhouse.

**Figure 15:**
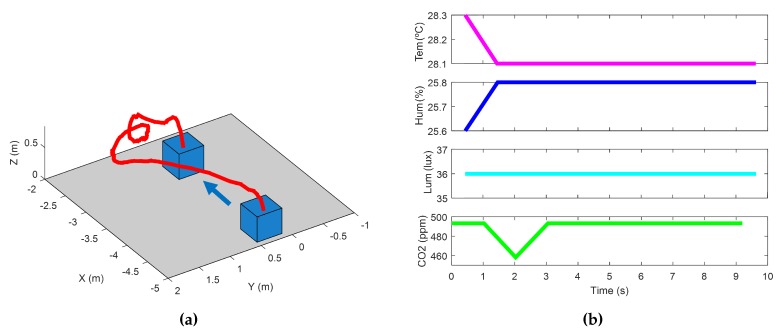
An example of the measurement of environmental variables from the UAV: (**a**) Paths of ground and aerial robots; (**b**) Evolution of environmental variables during the flight.

Finally, the aerial robot was used to perform short flights measuring the environmental variables around the ground robot. It should be reminded that the control of the aerial vehicle was manual, since the algorithms to navigate autonomously are under development and had not been already implemented at the time this work was presented. With this flight, we intended to prove that flying inside a greenhouse and mapping environmental variables with boarded sensors were possible. An example of flight is shown in Figure 15, where the mini-UAV takes off from the UGV, flies to a certain point and lands on the UGV, needing around 10 s. As shown, the values of environmental variables did not vary much in this short flight.

## 6. Conclusions

This paper proposes a multi-robot system to measure the environmental variables of interest in greenhouses. The robot team consists of a ground robot, which provides the fleet with autonomy and robustness, and an aerial robot, which contributes to the team with agility and speed. The robots are equipped with sensors of temperature, humidity, luminosity and carbon dioxide concentration, relevant variables for controlling and monitoring the conditions of crops.

Additionally, the paper addresses some collateral challenges of multi-robot systems, such as mission planning or guidance, navigation and control. The mission control and monitoring is performed by using a mission model, which is obtained from the experience of previous missions by means of process mining algorithms. The proposal of navigation for ground robots consists of two steps: the first time, the robot is teleoperated to build a map by a SLAM algorithm, and the next times, the robot navigates autonomously with an AMCL algorithm. The aerial robot is controlled manually due to the complexity of this environment, but autonomous navigation is proposed for future works.

A set of simulations of multi-robot system is performed in greenhouses with different configurations. The results show that the intervention of aerial robots potentially improves the performance of ground robot between 8% and 23%. In fact, the UAV is fundamental in the scenarios where the UGV cannot access several measure points.

Finally, a complete set of experiments was developed in an educational greenhouse. This experimental greenhouse has an irregular layout in contrast to productive greenhouses, so the algorithms had to work under harder conditions. The experiments validated both the mapping and navigation algorithms of the ground robot. Additionally, the maps of environmental variables built from this robot showed consistent results. Finally, the aerial robot was used to perform short flights in the greenhouse, taking off from the UGV, flying around to take several measurements and landing back on the UGV. It must be remarked that those flights were carried out by manual control, although our intention is that in future works, they will be performed autonomously.

## Figures and Tables

**Table 1:** Complete relevant state of the art of the work.

Work	System	Application	Variables	Scenario
Mandow et al. (1996) [35]	Large UGV	Spraying	None	Greenhouse
Sammons et al. (2005) [6]	Medium UGV	Spraying	None	Greenhouse
Belforte et al. (2006) [7]	Manipulator	Spraying	None	Greenhouse
Henten et al. (2009) [36]	Rail robot with manipulator	Harvesting	None	Greenhouse
Correl et al. (2009) [37]	Small UGV with manipulator and fixed sensors	Monitoring, watering inventorying and harvesting	Temperature and humidity	Garden
Pawlowski et al. (2009) [3]	WSN	Monitoring and control	Air temperature air humidity, PAR radiation and CO_2_ concentration	Greenhouse
Valente et al. (2011) [38]	WSN and small UAV	Monitoring	Temperature, humidity and solar radiation	Outdoor agriculture
Sánchez-Hermosilla et al. (2013) [39]	Large UGV	Spraying, pruning and harvesting	None	Greenhouse
Chung et al. (2014) [10]	Rail robot with manipulator	Inspection	Images	Greenhouse
Ko et al. (2015) [8]	Medium UGV	Spraying	None	Greenhouse
Roldán et al. (2015) [40]	Small UAV	Monitoring	Air temperature, humidity, luminosity and CO_2_ concentration	Greenhouse
Bengochea-Guevara et al. (2016) [41]	Medium UGV	Inspection and spraying	Images	Outdoor agriculture
Conesa et al. (2016) [42]	Team of UAVs and UGVs	Monitoring and spraying	Images	Outdoor agriculture
Ruiz-Larrea et al. (2016) [43]	Medium UGV	Monitoring	Ground temperature and humidity	Greenhouse
Roldán et al. (2016) [40]	Small UAV and medium UGV	Monitoring	Ground and air temperature and humidity	Greenhouse

**Table 2:** Comparison among ground and aerial units.

Robot	UGV	UAV
	(Robotnik Summit XL)	(Parrot AR.Drone 2.0)
**Specifications**		
Dimensions	722 mm × 610 mm × 392 mm	525 mm × 515 mm × 120 mm
Weight	45 kg	0.38 kg (1) 0.42 kg (2)
Speed	3 m/s	5 m/s
Autonomy	300 min	18 min
Charge	120 min	90 min
Load capacity	20 kg	0.2 kg
**Equipment**		
Cameras	PTZ and front cameras	Front and down cameras
Sensors	Temperature, humidity, luminosity and CO_2_	Temperature, humidity, luminosity and CO_2_

(1) Outdoor hull; (2) indoor hull.

**Table 3:** Sensor features. Source: datasheets.

Sensor	RHT03	TSL2561	MG811
Power supply	3.3–6.0 V	2.7–3.3 V	5.0 V and 7.5–12 V
Range	T: [−40; 80] °C	[0; 40,000] lux	[350; 10,000] ppm
	H: [0; 100]%		
Sensitivity	T: 0.1 °C/H: 0.1%	1 lux	Variable
Accuracy	T: 0.5 °C/H: 2%	Not available	Not available
Preparation time	0–5 s	<1 s	30–60 s
Response time	0–5 s	<1 s	15–30 s
Communications	Digital	I2C	Analog

**Table 4:** A fragment of the event log of a mission.

Case	Timestamp	Activity	Resource
MS01	30/04/2016-09:00:00	Begin	UGV
MS01	30/04/2016-09:00:02	Go to WP	UGV
MS01	30/04/2016-09:00:08	Arrive to WP	UGV
MS01	30/04/2016-09:00:40	Measure Temperature	UGV
MS01	30/04/2016-09:00:44	Measure Humidity	UGV
...	...	...	...

**Table 5:** Measurement frequencies according to greenhouse sizes.

Size of Greenhouse	Time of UGV	Time of Team	Savings
3600 m${}^{2}$ (60 m × 60 m)	2256 s	1967 s	12.79%
4200 m${}^{2}$ (70 m × 60 m)	2599 s	2260 s	13.05%
4800 m${}^{2}$ (80 m × 60 m)	2940 s	2554 s	13.12%
4900 m${}^{2}$ (70 m × 70 m)	2964 s	2632 s	11.20%
5400 m${}^{2}$ (90 m × 60 m)	3285 s	2870 s	12.62%
5600 m${}^{2}$ (80 m × 70 m)	3358 s	2978 s	11.30%
6000 m${}^{2}$ (100 m × 60 m)	3630 s	3164 s	12.84%
6300 m${}^{2}$ (90 m × 70 m)	3759 s	3337 s	11.24%
6400 m${}^{2}$ (80 m × 80 m)	3786 s	3400 s	10.21%
7000 m${}^{2}$ (100 m × 70 m)	4146 s	3680 s	11.24%
7200 m${}^{2}$ (90 m × 80 m)	4222 s	3804 s	9.90%
8000 m${}^{2}$ (100 m × 80 m)	4661 s	4199 s	9.92%
8100 m${}^{2}$ (90 m × 90 m)	4689 s	4272 s	8.90%
9000 m${}^{2}$ (100 m × 90 m)	5187 s	4718 s	9.05%
10,000 m${}^{2}$ (100 m × 100 m)	5801 s	5227 s	8.32%

**Table 6:** Team performance according to greenhouse sizes and obstacles.

Size	Obstacles	Duration	UGV/UAV Battery
Size of Greenhouse	Time of UGV	Time of Team	Savings
3600 m${}^{2}$ (60 m × 60 m)	2900 s	2252 s	22.35%
4200 m${}^{2}$ (70 m × 60 m)	3338 s	2596 s	22.22%
4800 m${}^{2}$ (80 × 60 m)	2940 s	2554 s	13.12%
4900 m${}^{2}$ (70 m × 70 m)	3711 s	2967 s	20.02%
5400 m${}^{2}$ (90 m × 60 m)	4232 s	3278 s	22.53%
5600 m${}^{2}$ (80 m × 70 m)	4199 s	3364 s	19.87%
6000 m${}^{2}$ (100 m × 60 m)	4666 s	3627 s	22.27%
6300 m${}^{2}$ (90 m × 70 m)	4704 s	3750 s	20.29%
6400 m${}^{2}$ (80 m × 80 m)	4616 s	3786 s	17.99%
7000 m${}^{2}$ (100 m × 70 m)	5180 s	4147 s	19.93%
7200 m${}^{2}$ (90 m × 80 m)	5160 s	4226 s	18.09%
8000 m${}^{2}$ (100 m × 80 m)	5699 s	4660 s	18.24%
8100 m${}^{2}$ (90 m × 90 m)	5634 s	4689 s	16.76%
9000 m${}^{2}$ (100 m × 90 m)	6222 s	5184 s	16.68%
10,000 m${}^{2}$ (100 m × 100 m)	6744 s	5698 s	15.51%

## Abbreviations

The following abbreviations are used in this manuscript:

MIMO: Multiple-Input Multiple-Output
WSN: Wireless Sensor Network
UGV: Unmanned Ground Vehicle
UAV: Unmanned Aerial Vehicle
GNC: Guidance Navigation and Control
MIMO: Multiple-Input Multiple-Output
PID: Proportional, Integral and Derivative
PAR: Photosynthetically Active Radiation
IMU: Inertial Measurement Unit
GNSS: Global Navigation Satellite System
I2C: Inter-Integrated-Circuit
SDA: Serial Data Line
SCL: Serial Clock Line
ADC: Analog to Digital Converter
WP: Waypoint
ROS: Robot Operating System
EKF: Extended Kalman Filter
AMCL: Augmented Monte Carlo Localization
SLAM: Simultaneous Location and Mapping

## Appendix A. Multimedia Extensions

The following link shows a video that illustrates the experiments developed for this work: https://youtu.be/o6SXPQv9LyU.
